# Engagement With and Acceptability of Digital Media Platforms for Use in Improving Health Behaviors Among Vulnerable Families: Systematic Review

**DOI:** 10.2196/40934

**Published:** 2023-02-03

**Authors:** Elisabet V Eppes, Marycatherine Augustyn, Susan M Gross, Paris Vernon, Laura E Caulfield, David M Paige

**Affiliations:** 1 Department of Population, Family, and Reproductive Health Bloomberg School of Public Health Johns Hopkins University Baltimore, MD United States; 2 Department of International Health Bloomberg School of Public Health Johns Hopkins University Baltimore, MD United States

**Keywords:** text messaging, social media, mobile app, low-income, engagement, health promotion, community, nutrition and physical activity, pregnancy, breastfeeding, maternal and child health, mobile phone

## Abstract

**Background:**

The use of digital communication platforms to improve health behaviors has increased dramatically over the last decade. Public health practitioners have adopted digital communication technologies such as text messages, mobile apps, and social media to reach diverse populations. However, the effectiveness of digital communication platforms used by community-serving agencies remains unclear, and patterns of engagement and acceptability of different platforms have not been studied.

**Objective:**

This review aimed to identify the types of digital communication strategies used by community-serving organizations to promote healthy behaviors, assess the strength of evidence for health behavioral change, and describe the degree of consumer engagement with and acceptability of these strategies. The study population included low-income pregnant women, parents of young children, and adolescents.

**Methods:**

A systematic review was conducted according to the PRISMA (Preferred Reporting Items for Systematic Reviews and Meta-Analyses) guidelines using PubMed, Scopus, Web of Science, CINAHL, and APA PsycInfo, covering research conducted from 2009 to 2022. Studies were included if they examined the use of digital communication (ie, texting, mobile apps, or social media) to promote healthy behaviors in the target population. Risk of bias and strength of evidence were assessed using the Effective Public Health Practice Project Risk of Bias tool and criteria from Agency for Healthcare Research and Quality, respectively.

**Results:**

Twenty-three peer-reviewed research studies published between 2012 and 2022, conducted in the United States, the United Kingdom, and Australia, were included in the review. The sample comprised studies exploring the use of texting (n=12), apps (n=6), social media (n=3), and multiple platforms (n=2; eg, texting and mobile apps). Targeted health behaviors included healthy diet, physical activity, obesity prevention, healthy pregnancy, breastfeeding, vaccine use, smoking cessation, and nutrition benefit redemption. The sample included 8 randomized controlled trials, 6 pretest-posttest design, 3 mixed methods studies, 2 pilot studies, 1 feasibility study, 1 prospective cohort study, 1 descriptive study, and 1 cross-sectional study. The median sample size was 77.5. There was no strong evidence to suggest the effectiveness of digital media campaigns in improving health behaviors; however, there were moderate to high levels of engagement and high levels of acceptability across digital platforms.

**Conclusions:**

Low-income pregnant women, parents of young children, and adolescents demonstrated moderate levels of engagement with and high levels of acceptability of digital media health campaigns conducted by community-serving agencies. The effectiveness of these strategies in improving health behaviors was inconclusive. Additional rigorous studies with larger sample sizes are required. In addition, more research is required to consistently measure and report participants’ engagement with each platform. Digital communication platforms are critical tools for public health practitioners, and future investigations of the effectiveness of these platforms in engaging clients and improving health behaviors will maximize client services.

## Introduction

### Background

The use of digital communication platforms to improve health behaviors has expanded dramatically in the last decade. Digital media and marketing technologies such as SMS or text messages, mobile apps, and social media are being leveraged to reach diverse populations in ways that are efficient, convenient, and engaging [1-4]. These technologies are being used to promote healthy behaviors by answering client questions in real time, monitoring health behaviors, and providing education and support [4-7]. Digital interventions are often low cost and relatively easy for public health organizations to implement compared with in-person interventions [8]. They have frequently been used to target specific populations, including young people (because they are the most frequent users of smartphones and social media platforms) and low-income individuals who may not have consistent access to high-quality health information and may struggle with access to in-person preventive health services owing to transportation challenges, lack of childcare, and other barriers [1,9-13].

Digital communication campaigns have been leveraged to improve nutrition and physical activity (PA), increase breastfeeding and immunization rates, reduce barriers to preventive health screenings, and improve access to mental health support [14-17]. Petkovic et al [14] reported that social media interventions designed to increase PA may be effective, and social media interventions may improve overall well-being. However, the review indicated that there was insufficient evidence for improvement in other outcomes, including breastfeeding, condom use, diet quality, medication adherence, medical screening and testing, tobacco use, and vaccination. Welch et al [15] provided an overview of systematic reviews of social media intervention studies targeting a broad range of health behaviors and reported mixed effects on health behaviors and outcomes. The authors reported that social media interventions are more effective among disadvantaged populations such as youth, older adults, low-income people, and people residing in rural areas. Richardson et al [16] reported that SMS text messaging is an effective tool to influence caregiver behavior and child outcomes in health, whereas the results of the studies reviewed by Poorman et al [17] were mixed, and the authors concluded that SMS texting interventions that are based on an established theory of behavior change and use motivational language, as opposed to informational language, are more likely to be successful.

To date, no reviews have examined the use of different digital communication platforms to improve health behaviors among vulnerable families. Accordingly, this review focused on the use of diverse digital health promotion campaigns by community-serving agencies to reach low-income pregnant women, parents of children aged <18 years, and adolescents living in high-income, predominantly English-speaking countries. Members of the selected population share demographic, income, and health characteristics in addition to their use of community support services. For the purposes of this study, community-serving agencies included health departments; local nutrition agencies such as the Supplemental Nutrition Assistance Program and Special Supplemental Nutrition Program for Women, Infants, and Children (WIC); food pantries; public schools; home-visiting programs; and early childhood education programs. Considering the extensive use of digital technology among young parents and adolescents; the key developmental periods associated with pregnancy, childhood, and adolescence; and the barriers associated with accessing preventive health services in person, digital communication platforms are particularly relevant for this population [17-19].

Although existing reviews have focused on the correlation between the use of these technologies and health behavior change, this review also incorporated an investigation of the degree of engagement with and acceptability of these platforms [20]. It is important to explore technologies that elicit the greatest engagement from members of this population, as well as those that are the most acceptable, because these are important factors in the relationship between digital technologies and behavior change and could provide insights into the inconsistent impacts of digital health promotion campaigns on health behaviors [20-24].

### Objective

The objective of this review was to identify the types of digital communication strategies used by community-serving organizations to promote healthy behaviors among vulnerable families, assess the strength of evidence for health behavior change, and describe the degree of engagement with and acceptability of various strategies.

## Methods

### Search Methodology

The PRISMA (Preferred Reporting Items for Systematic Reviews and Meta-Analyses) guidelines were followed in this review [25]. The PRISMA checklist is available in Multimedia Appendix 1.

### Information Sources

Literature searches were conducted using 5 electronic publication databases (PubMed, Scopus, Web of Science, CINAHL, and APA PsycInfo). In addition, the lists of references of relevant systematic reviews and reports were searched for studies relevant to the review.

### Study Eligibility Criteria

This review used a systematic approach to retrieve the relevant research studies and included all study designs for identifying the wide range of evidence examining the use of digital communication (ie, texting, mobile apps, or social media) to promote health behavior change among the target population. The inclusion criteria are detailed in Textbox 1. For the purposes of this review, program participation and use of program components were considered health behaviors. Studies that discussed industry marketing were excluded.

**Inclusion criteria.**

**Topic**

- Use of digital health promotion to target low-income populations, including text messages, mobile apps, or social media

**Participants**

- Parents or caretakers, women of reproductive age, adolescents, or children

**Settings**

- Community-serving agencies in high-income, English-speaking countries (the United States, Canada, the United Kingdom, or Australia)

**Language**

- English

**Publication date**

- January 2009 to March 2022

### Types of Participants

The participants were low-income parents and caregivers of children or adolescents, pregnant women, and adolescents participating in community-serving programs.

### Search Strategy

The initial search was performed between June 2020 and September 2020. A subsequent search was conducted in April 2022 to ensure the inclusion of recent publications. Search terms relevant to 3 distinct concepts were used, as outlined in Textbox 2. All titles and abstracts were initially screened to ensure relevance, no duplication of articles, and adherence to the inclusion criteria.

**Search terms.**

**Concept 1**

- “Food Assistance” OR “Food Aid Program” OR “Healthy People Programs” OR “Health Promotion” OR “WIC Program” OR “SNAP Program” OR “Wellness Program” OR “Health Campaign.”

**Concept 2**

- “Vulnerable Populations” OR “Poverty” OR “Underserved Population” OR “Disadvantaged” OR “Low-Income Population.”

**Concept 3**

- “Marketing of Health Services” OR “Digital Divide” OR “Mobile Applications” OR “social media” OR “text message” OR “Communication Technology.”

### Data Extraction

**Figure 1:**
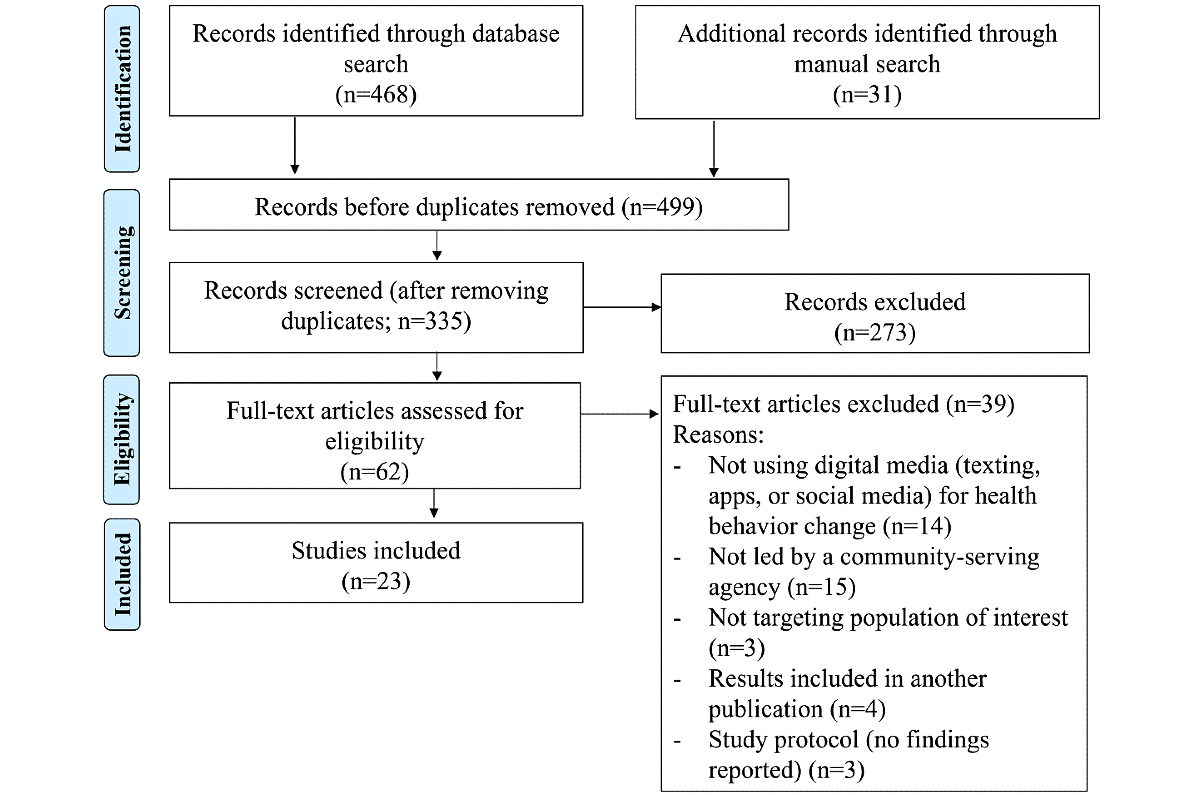
Flow diagram of the systematic review.

The retrieved studies were imported into the screening and data extraction tool developed by Covidence, an Australian nonprofit [26], for title and abstract screening, full-text review, and extraction. Of the 335 nonduplicated records, 62 (18.5%) articles were subjected to full-text review by 2 authors to determine eligibility (Figure 1). The reasons for excluding studies at the full-text review stage included the following: the study did not use digital communication to improve health behaviors; the study was not led by a community-serving agency; the study did not target the population of interest; and the results of the study were published elsewhere. A total of 23 articles were eligible for data abstraction and were included in this review.

### Risk of Bias Assessment, Effect Measures, Data Synthesis, and Analysis

The purpose of the digital campaign was first identified using an emergent process. The broad categories of purposes included health promotion and education, support, and behavior monitoring; more specific categories were determined during the analysis and are described in the *Results* section.

The degree of participant engagement and acceptability associated with each digital health promotion campaign was then assessed. In digital media studies, some forms of engagement can be measured across different platforms, whereas others are specific to a particular platform. For example, enrollment can be assessed for texting, mobile apps, and social media; responding can be assessed for texting and apps; clicking can be assessed for texting (of embedded links) and social media; and frequency of use can be assessed for apps and social media. Platform-specific assessments include reading (texting only), comments (social media only), *likes* (social media only), shares (social media only), and the use of app components (mobile apps only). To account for the differences in measurements among the platforms, the authors first categorized the studies by platform and then conducted an analysis of engagement. Across the platforms studied, acceptability measures included asking users about digital campaign usefulness, convenience, understandability, and enjoyability.

Finally, the quality of the health behavior evidence described in the studies was appraised by 4 researchers. The risk of bias was determined using the Effective Public Health Practice Project Risk of Bias tool [27] and included measurements for selection bias, confounders, reliable data collection tools and procedures, sample retention (ie, attrition rates), and protocol integrity. The strength of evidence assessments were conducted using criteria from the Agency for Healthcare Research and Quality [28], which included study limitations, directness of intervention, consistency and precision of implementation, reporting bias, and risk of bias. Each study was assessed independently by 2 researchers, first for risk of bias and then for strength of evidence. Any disagreements in assessments were reconciled by a third researcher.

## Results

### Overview

The sample of this review consisted of 23 academic studies published between 2012 and 2022. Most (21/23, 91%) studies were conducted in the United States; 1 study was conducted in the United Kingdom, and 1 in Australia.

Of the 3 digital media platforms under review, the sample included 12 (52%), 6 (26%), 3 (13%), and 2 (9%) studies exploring the use of texting, mobile apps, social media, and multiple platforms (eg, texting and mobile apps), respectively. Of the 12 texting studies, 5 (42%) explored the use of one-way texting (ie, no opportunity for participants to respond), whereas 7 (58%) used a two-way texting campaign (participants could respond to investigators). Of the 23 studies, 1 (4%) study used both social media and an app, and 1 (4%) used both texting and an app. The social media studies focused on Facebook (3/23, 13%) and Twitter (1/23, 4%). The targeted health behaviors included healthy diet, PA, healthy weight and obesity prevention (9/23, 39%), healthy pregnancy (5/23, 22%), breastfeeding (4/23, 17%), vaccine use and disease prevention (2/23, 9%), smoking cessation (1/23, 4%), and WIC benefit redemption (1/23, 4%). Overall, 4% (1/23) of studies did not focus on health behavior but explored the feasibility of using social media to communicate with low-income mothers participating in a home-visiting program [29]. Community-serving agencies included WIC (12/23, 52%), health departments (2/23, 9%), food pantries (1/23, 4%), K-12 schools (2/23, 9%), public housing (1/23, 4%), home visits (1/23, 4%), Healthy Start (1/23, 4%), and a combination thereof (3/23, 13%).

The 23 study designs included 8 (35%) randomized controlled trials (of which 3, 37% were randomized controlled pilot studies), 6 (26%) pretest-posttest design, 3 (13%) mixed methods studies, 2 (9%) pilot studies, 1 (4%) feasibility study, 1 (4%) single-sample prospective cohort study, 1 (4%) descriptive study, and 1 (4%) cross-sectional study. The sample sizes ranged from 14 [30] to 30,440 [31] with a median of 77.5. The study population consisted primarily of reproductive-aged women (12/23, 52%), including those who were pregnant or mothers, caregivers of infants and young children (7/23, 30%), and adolescents (2/23, 9%). One of the 23 studies (4%) focused on mother and child dyads, wherein the child was 9-14 years old. [32]. The following data for each study are presented in Table 1: digital media platform, study reference, targeted health behavior, study objective, sample size, community agency, location, study design, theoretical underpinnings, description of the intervention, and duration of the study.

Each digital campaign could have one or more purposes, including general health messages, individually tailored messages, reminders, client support, or behavior monitoring. Individually tailored messages were sent to study participants based on certain characteristics, such as trimester of pregnancy, whereas general health messages were sent to all study participants regardless of their individual characteristics. General health message topics included healthy pregnancy, breastfeeding, healthy diet, fruit and vegetable (FV) consumption, PA, obesity prevention, vaccine use, and disease prevention, whereas individually tailored message topics included healthy pregnancy, smoking cessation, breastfeeding, and healthy weight. Campaigns whose purpose included sending reminders prompted study participants to engage in certain health behaviors at specific times. The remaining topics included health care appointments, healthy diet, and PA. Campaigns centering on support used the digital platforms to assist study participants with a particular health goal, such as smoking cessation, healthy diet and PA, breastfeeding, healthy pregnancy, disease prevention, or engagement in a home-visiting program. Finally, behavior monitoring involved the use of the digital platform to track health behaviors and outcomes, including PA and body weight.

The purpose of the digital campaigns included general health messages only (8/23, 35%), individually tailored messages only (2/23, 9%), general health messages and reminders (1/23, 4%), general health messages and support (3/23, 13%), general health messages and behavior monitoring (2/23, 9%), individually tailored messages and support (2/23, 9%), individually tailored messages and behavior monitoring (1/23, 4%), general health messages, reminders, and support (1/23, 4%), individually tailored messages, reminders, and support (1/23, 4%), and general health messages, reminders, support, and behavior monitoring (2/23, 9%; Multimedia Appendix 2). Of the 23 campaigns, 17 (74%) campaigns used general health messages, 6 (26%) used individually tailored messages, 5 (22%) sent reminders, 9 (39%) provided support, and 5 (22%) conducted behavior monitoring. Two-way texting campaigns were much more likely to be designed to provide support (6/23, 26%) than one-way texting campaigns (1/23, 4%). Behavior monitoring was conducted using mobile apps (3/23, 13%) and two-way texting (2/23, 9%).

Engagement with and acceptability of the platforms, as reported in 87% (20/23) of the studies, were concrete indicators of study participant behavior and are important mediators of health behavior change. Engagement and acceptability results are reported in the following sections, followed by health behavior change outcomes. Multimedia Appendix 3 includes a summary of engagement, acceptability, and health behavior outcomes.

**Table 1:** Characteristics of included studies categorized by digital media platform.

Type of digital platform and study reference		Targeted health behavior	Study objective	Sample size, community agency, location, and population characteristics	Study design	Theory	Intervention and duration
**One-way texting**							
	Evans et al [33]	Healthy pregnancy	To evaluate understandability, satisfaction, and behavior change potential of *text4baby*	86 low-income pregnant and postpartum women (38 control; 48 intervention); agency: Fairfax County Health Department, Virginia; 80% participants were of Hispanic origin; average age 28 years	Randomized pilot evaluation study	HBM^a^, SCT^b^, and TTM^c^	3 weekly texts (for 2-3 months) with developmentally sensitive tips for improving prenatal and postpartum health outcomes
	Gazmararian et al [34]	Healthy pregnancy	To assess factors related to enrollment process and reception of *text4baby* platform	468 pregnant and postpartum women enrolled in WIC^d^ in Atlanta, Georgia; 54% aged <25 years, 26% ≥30 years; 91% Black individuals; 42% at least some college education; 82% household income ≤US $20,000	One sample prospective cohort (surveys)	Not reported	3 weekly texts (for 2 months) with tips for improving prenatal and postpartum health outcomes targeted to the point in the pregnancy or the age of child
	Holmes et al [35]	Healthy pregnancy	To investigate the effect of educational texts on excessive GWG^e^ in an overweight or obese population	83 (42 intervention and 41 control) pregnant WIC clients in Oahu, Hawaii; mean age 28, SD 5 years; 65.5% Native Hawaiian, Pacific Islander or American Indian individuals; 54.8% had some college education or more	RCT^f^	SCT	Intervention group received texts on nutrition and PA^g^ during pregnancy. Control group received texts about general health topics. Both groups received 1 text/week for 18 weeks.
	Power et al [36]	FV^h^ consumption	To examine efficacy and acceptability of *Txt4Happy-Kids*, which promotes FV intake among families	72 parents of young children recruited from Head Start, WIC, public library, and a free family health fair in Fairbanks, Alaska; majority White females 25-34 years of age with some college education and a child <5 years old	Pretest-posttest design study using surveys	SCT	11-week intervention that sent parents twice weekly texts encouraging them to serve more FV to their child
	Tagai et al [37]	Smoking cessation	To examine feasibility of a text message-based smoking cessation intervention for urban, underserved postpartum women	43 women enrolled in 15 WIC clinics in Philadelphia, Pennsylvania; mean age 29 years; 63% Black and 12% White individuals	Single arm feasibility, convergent mixed methods	C-SHIP^i^ model	Text messages tailored to needs of low-income postpartum women who had made a quit attempt during pregnancy provided support during times of smoking lapse or craving. Upon giving birth, participants received 3 texts/day for 1 month.
**Two-way texting**							
	Banna et al [38]	Breastfeeding and infant weight	To describe development, implementation, and acceptability of texts to improve feeding practices and prevent excessive weight gain in infants	202 (100 control, 102 intervention) parents or caregivers of infants enrolled in WIC in Puerto Rico and Hawaii; mean age 27 years; 54% had some college education or more; 60% Hispanic and 7%-10% Native American individuals	Feasibility study of an RCT	TTM	Weekly text messages for 4 months (control: text messages about general infant health issues; intervention: messages for improving infant feeding practices)
	Palacios et al [39]	Banna et al [38]	To test effectiveness of texts at improving feeding practices and infant weight	Banna et al [38]	Multisite RCT (follow-up to Banna et al) [38]	Banna et al [38]	Banna et al [38]
	Griffin et al [40]	Healthy diet and PA	To evaluate changes in dietary and PA behaviors and weight after implementation of a text messaging initiative (*MyQuest*) among obese or overweight women	104 low-income women who were clients of SNAP^j^, Department of Housing and Urban Development, or Alabama Food Bank; average age 36 years; 54% African American individuals; 32% had a high school diploma or GED	Pretest-posttest design surveys	SCT	Texts were scheduled for daily delivery at 3 periods of the day (for 12 weeks) and included tips on diet and exercise, web links, goal-setting prompts, and reminders or questions about exercise or healthy eating that required a response. Daily step counts and weekly body weights were collected through text responses.
	Griffin et al [41]	Griffin et al [40]	To determine whether text messaging is an effective education and support tool to help women trying to lose weight	Griffin et al [40]	Griffin et al [40]	Griffin et al [40]	Griffin et al [40]
	Harari et al 2017 [42]	Breastfeeding	To test acceptability and feasibility of *LATCH*^k^ intervention	52 pregnant women (30 intervention, 22 control) enrolled in 2 WIC sites in Connecticut; mean age 26 years; 75% Hispanic, 17% African American, and 6% White individuals	Randomized controlled pilot study	Theory of planned behavior	Web‐based texting intervention providing evidence‐based breastfeeding education through automated texts (for 12-24 weeks) and a mode for pregnant women and mothers to exchange texts with breastfeeding peer counselors
	Martinez-Brockman et al 2017 [43]	Breastfeeding	To describe engagement with *LATCH* and assess association between engagement and exclusive breastfeeding	70 pregnant women enrolled in the WIC breastfeeding peer counselor program at 1 of 4 WIC sites in Connecticut; average age 27 years; 75% Hispanic individuals; 40% had more than a high school education	RCT	HAPA^l^	Messages designed for each trimester of pregnancy and up to 2 weeks postpartum were sent automatically using a predetermined schedule (for ≥14 weeks)
	Song et al [44]	Healthy pregnancy	To gauge ease of use, coverage, and real-life consequences of texts to distribute pregnancy and health-related information	20 WIC-eligible pregnant minority women who were clients of prenatal programs at the Milwaukee Health Department, Wisconsin; mean age 21 years	Pretest-posttest design; mixed methods	Not reported	One-month intervention involving use of cell phones by participants to text pregnancy-related questions. Participants received either a direct answer or encouragement to seek answers from health care providers.
**Mobile app**							
	Clarke et al 2018 [32]	FV consumption	To test effectiveness of *VeggieBook* smartphone app in increasing use of vegetables in meal prep and gauge app use	289 (106 control, 183 experimental) household cooks and one of their 9-14-year-old children recruited from food pantries in Los Angeles County, California; on average, moms had between a middle and high school education level and were in their late 30s	RCT	Not reported	Provision of experimental families with a smart phone with app and 3-month data plan. App offers vegetable-based recipes, food tips, and strategies for making mealtimes healthier. Users customize materials to meet preferences. Control and experimental groups received supplemental vegetables weekly for 4 weeks.
	Gilmore et al [45]	Healthy weight	To test efficacy of a mobile app, *SmartLoss*, to promote postpartum weight loss	40 overweight or obese postpartum mothers participating in WIC in Baton Rouge, Louisiana; mean age 26 years; 74% African American individuals	Randomized controlled pilot study	Not reported	Weight-loss lifestyle app tailored to postpartum women—real-time weight and activity monitoring, scheduled delivery of health information, and interventionist feedback. Intervention lasted 4 months.
	Hull et al [46]	Dietary quality, childhood obesity prevention	To test *CHEW*^m^ app, focusing on use, usability, and perceived barriers and benefits	Mothers of 63 Black and Hispanic children aged 2-4 years old participating in WIC in Tennessee; the largest age group for mothers was 25-34 years	Pilot test	Socioecological model	Goals of app were to make WIC shopping experience easier, maximize WIC benefit redemption, and improve parent snack feeding practices. App prototype included WIC Shopping Tools and nutrition education. App was tested for 3 months.
	Nollen et al [47]	Obesity prevention	To test a mobile technology intervention for use and estimate effect sizes for a fully powered trial	51 low-income, racial or ethnic-minority girls aged 9-14 years (26 intervention, 25 control) participating in after-school programs in Kansas; average age 11 years; 84% African American and 8% Hispanic or Latina individuals	RCT	Behavioral weight control principles	*MyPal* handheld computer comparable in size, weight, and appearance to a smartphone which included goal setting and planning. Intervention lasted 12 weeks.
	Reyes et al [48]	Disease prevention	Explore women’s views on mHealth^n^ app, *Ever-healthier Women*, to seek health information and inform health choices	15 low-income women who used WIC services in a large Northeastern US city; average age 25 years; 13/15 were African American individuals	Qualitative descriptive study	SCT	mHealth app designed to provide women with easy access to preventive health information and promote adherence to potentially life-saving clinical screenings and disease-prevention behaviors. Intervention lasted 3 weeks.
	Zhang et al [31]	WIC benefit redemption	To examine relationship between use of mobile app *WICShopper* and redemption of prescribed food packages	30,440 WIC HH^o^ in West Virginia; 88% of HH were non-Hispanic White individuals	Mix of cross-sectional and longitudinal study designs	Not reported	Purpose of app is to facilitate benefit redemption via multiple features, such as checking benefit balance and scanning the bar code to check eligibility. Study period was 12 months.
**Social media**							
	Allen et al [49]	HPV^p^ vaccine use	To assess feasibility of a Twitter campaign to promote knowledge about HPV vaccine	35 women residing in low-income, public housing in Massachusetts; mean age 22 years; 61% had at least some college education; 51% Black individuals; 40% born outside United States	Feasibility study, pretest-posttest design surveys	HBM	Daily tweet over a period of 1 month addressing HPV vaccination and promoting cervical cancer screening
	Dion [29]	Health communication	To present experience in use of Facebook between health visiting team and clients	250 low-income families with young children in Poole, United Kingdom participating in a home-visiting program; average age of mothers with first baby is 23 years; 74% White British individuals	Pilot study, posttest survey	Not reported	Facebook page with private messaging service for clients and public page with general information. Study period was 5 years.
	Zhang, Panichelli, and Hall [50]	Healthy eating	To assess impact of *CM*^q^ Facebook page on healthy eating behaviors	397 caregivers enrolled in WIC with a child aged ≤5 who never previously followed CM on Facebook from various US states; At follow-up, most caregivers were in the 25-34 years age group; most had at least some college; 41% non-Hispanic White, 15% non-Hispanic Black, and 41% Hispanic individuals	Single group pretest-posttest design	SCT	Use of Facebook page over 2 months. Page publishes recipes, tips for food planning and cooking easy meals on a limited budget, videos, and live events.
**Multiple: mobile app and social media**							
	Koorts et al [51]	PA	To evaluate acceptability, feasibility, impact, engagement, and adherence to *RAW-PA*^r^ app	142 adolescents attending schools in low SES^s^ areas of Melbourne, Australia; mean age 14 years	Mixed methods (building on previous RCT)	SCT and behavioral choice theory	RAW-PA was a 12-week cluster RCT conducted in 2016-2018 studying use of wearable technology to increase PA among adolescents. Intervention combined wearable activity tracker, app promoting awareness of PA, and social media–based behavior change resources.
**Multiple: mobile app and two-way texting**							
	Foster et al [30]	Healthy pregnancy and prevention of adverse birth outcomes	To test acceptability and feasibility of an interactive mHealth app	14 African American women in various reproductive stages enrolled in Healthy Start in Atlanta, Georgia; average age 22 years; most had some high school or in process of obtaining GED^t^	Pilot testing, mixed methods, and CBPR^u^	Not reported	App was designed to be interactive; participants received texts and were prompted to respond to texts according to their assigned profile. Women in each profile received questions on a daily, weekly, or monthly schedule via text over a period of 11 weeks.

^a^HBM: Health behavior model.

^b^SCT: Social Cognitive Theory.

^c^TTM: transtheoretical model.

^d^WIC: Special Supplemental Nutrition Program for Women, Infants, and Children.

^e^GWG: gestational weight gain.

^f^RCT: randomized controlled trial.

^g^PA: physical activity.

^h^FV: fruit and vegetable.

^i^C-SHIP: Cognitive Social Health Information Processing.

^j^SNAP: Supplemental Nutrition Assistance Program.

^k^LATCH: Lactation Advice thru Texting Can Help.

^l^HAPA: Health Action Process Approach.

^m^CHEW: Children Eating Well.

^n^mHealth: mobile health.

^o^HH: household.

^p^HPV: Human Papilloma Virus.

^q^CM: Cooking Matters.

^r^RAW-PA: Raising Awareness of Physical Activity.

^s^SES: socioeconomic status.

^t^GED: graduate equivalency diploma.

^u^CBPR: community-based participatory research.

### Engagement Outcomes

#### Texting

The study by Gazmararian et al [34] was the only (1/5, 20%) one-way texting campaign study to measure engagement. In this study, examining the *text4baby* platform, which sends texts with tips for improving prenatal and postpartum health outcomes targeted to the point in the pregnancy or the age of the child, 51% of study participants attempted self-enrollment; 69% who attempted successfully enrolled; 92% regularly read all messages; and 88% planned to continue enrollment.

Overall, 71% (5/7) of the two-way texting studies measured engagement (defined as reading and responding to texts). In the study by Banna et al [38], which studied the use of text messages to improve infant feeding, 52% to 54% of individuals responded to the first 4 text questions that were sent, and 32% to 34% responded to the final 3 text questions. In the study by Griffin et al [41], of the study participants who completed the texting program related to healthy diet and PA, 70% responded to text messages before and after the intervention. Approximately 82% of participants in the study by Harari et al [42] reported that they “*always*” or “*very often*” read the text messages related to breastfeeding during the prenatal and postpartum periods; 90% texted an original comment or question (other than “ok” or “thanks”); and 77% clicked on at least one weblink sent via text. In the study by Martinez-Brockman et al [43], participants responded to an average of 27% of text messages related to breastfeeding during the prenatal and postpartum periods. The participants clicked on an average of 22% of the possible videos, weblinks, and photos. Participants in the study by Song et al [44] texted an average of 3.5 questions per week related to healthy pregnancy.

#### Apps

All (6/6, 100%) of the app studies reported on engagement outcomes. Most intervention households in the study by Clarke et al [32] created 15 pieces of content (“Veggie-Books”) in the *VeggieBook* app, which aims to increase FV intake. Parents, on an average, used the 2 main app components approximately 5 times over the course of the 10-week study. In the study by Gilmore et al [45], participants used the weight-loss lifestyle app tailored to postpartum women 4 times per week. Participants testing the *Children Eating Well (CHEW)* app in the study by Hull et al [46] used the app on an average of once a week over a 3-month period for approximately 4.5 minutes per session. The goals of *CHEW* were to make the WIC shopping experience easier, maximize WIC benefit redemption, and improve parent snack feeding practices. Participants in the study by Nollen et al [47] used the weight control app, *MyPal*, which included goal setting and planning, on 63% of days and responded to 42% of prompts. In the study by Zhang et al [31], 72% of households used the *WICShopper* app, which facilitates WIC benefit redemption, at least once during the study period. The app users, on an average, activated the app once a week. During follow-up, 56% of participants in the study by Reyes et al [48] were still using *Ever-healthier Women*, an app designed to provide women with easy access to preventive health information and promote adherence to potentially life-saving clinical screenings and disease-prevention behaviors.

#### Social Media

All (3/3, 100%) of the social media studies reported on engagement outcomes. In the study by Allen et al [49], 17% of participants *liked* the Human Papilloma virus vaccine–related tweets, and 11% of participants *retweeted* the messages. In the study by Dion [29], 61% of mothers who had a baby in the past year joined the health visiting program’s Facebook page, and 9% of clients responded with *likes*. In the study by Zhang et al [50], approximately 75% of study participants viewed the *Cooking Matters* Facebook page, which focused on healthy eating on a budget, at least a few times a week. Interaction with the Facebook page varied from 29% of followers who commented on posts to 59% of followers who reacted to posts (eg, likes).

#### Multiple Platforms

Both studies that explored multiple platforms reported on engagement outcomes. In the study by Koorts et al [51], approximately 85% of participants registered for the *Raising Awareness of Physical Activity (RAW-PA)* Facebook group. *RAW-PA* combined a wearable activity tracker, an app promoting awareness of PA, and social media–based behavior change resources to increase PA among adolescents. Engagement in the *RAW-PA* Facebook group declined over time, and only 19% of the participants used the app daily after the intervention. Foster et al [30] studied the use of a mobile app and text messaging to promote healthy pregnancies and found that the average response rate to the 3 highest response rate text messages in each reproductive stage was approximately 61%.

#### Engagement Summary

Enrollment ranged from 51% to 85% (mean 66%, 3 studies), whereas continued enrollment ranged from 19% to 88% (mean 54%, 3 studies). The percentage of participants who responded or sent original messages ranged from 70% to 90% (mean 80%, 2 studies), and the percentage of texts or prompts that elicited responses ranged from 27% to 61% (mean 43%, 4 studies). The percentage of participants who clicked on content in texts or an app ranged from 22% to 77% (mean 50%, 2 studies), whereas the proportion of participants who regularly read texts ranged from 82% to 92% (mean 87%, 2 studies). The 1 study that measured content creation within an app reported that households created an average of 15 pieces of content in the app during the study period. Frequency of use of an app, texting program, or social media page ranged from 0.5 times per week to 4 times per week (mean, 2 times/week; 7 studies). The percentage of participants who *liked* social media posts ranged from 9% to 59% (mean 28%, 3 studies), the percentage of participants who shared posts was 11% (1 study), and the percentage who commented was 29% (1 study).

### Acceptability

#### Texting

Overall, 60% (3/5) of the one-way texting studies provided results on acceptability. In the study by Gazmararian et al [34], 95% of the participants reported that participating in the texting campaign was easy. In the study by Power et al [36], which aimed to increase FV consumption through the *Txt4HappyKids* campaign, 79% of participants thought the texting program was very credible; 71% found the text messages very useful; 82% stated that they would recommend the program to a friend; and 76% felt that they received the appropriate number of messages. In the study by Tagai et al [37], 95% of participants found *TxT2Connect*, a smoking cessation campaign, helpful and enjoyed the messages; 5% of participants said that the program was not helpful, wishing that the “messages were more detailed” and stating that “in-person support would be more beneficial.”

Moreover, 57% (4/7) of the two-way texting studies reported on acceptability. In the study by Banna et al [38], which tested the use of weekly text messages to improve infant feeding practices, 70% of participants said that the texts were sent at acceptable times, were useful and not irritating, and easy to understand. In terms of what participants liked about the intervention, convenience was the most commonly cited, followed by the short length of the messages and the usefulness of the information received. In the study by Harari et al [42], 91% of participants reported that they would recommend the texting program to a friend. Song et al [44] reported that 80% of the participants reported that it was easy to text questions; 65% reported that information was easy to understand; and 65% reported that the texting system made finding information quick and easy. Participants also liked the fact that the system allowed for interactive dialogue. Griffin et al [40] reported that 58% of participants said the *MyQuest* texting intervention, which sought to promote healthy diet and PA, was helpful, and most said that the number of texts sent was appropriate. Participants generally found the text to be informative and motivating.

#### Apps

Overall, 50% (3/6) of the app studies reported on acceptability. In the study by Hull et al [46], participants who used the *CHEW* app rated the WIC Shopping Tools relatively high on usability and benefits. The Yummy Snack Gallery and Healthy Snacking Tips scored higher on usability than on benefits. Qualitative feedback from participants who chose not to use the app pointed to several barriers, including technical issues, lack of interest in the content, not remembering to use it, and not noticing alerts. In the study by Nollen et al [47], which reinforced app use through a song-based reward system that provided the adolescent study participants 1 song per day if they responded to 80% of daily prompts, the favorite parts of the app were obtaining songs (68%) and setting goals (36%). The least favorite part of the app was the reminder to self-monitor progress toward healthy eating and PA goals (32%). In the study by Reyes et al [48], all participants stated that they would recommend the app to their friends and families and thought that the app would be good for women of all ages and circumstances. All participants thought that the app was easy to use, liked using it, and had no privacy concerns or technical difficulties; 64% of participants thought the app was “simple” or “self-explanatory.”

#### Social Media

Overall, 9% (2/23) of social media studies reported on acceptability. In the posttest survey, 71% of participants in the study by Allen et al [49] agreed with the statement that “Twitter messages are a good way to educate women about the HPV vaccine.” In the study by Zhang et al [50], recipes were the most-liked content on the Facebook page, followed by tips on feeding children and tips on food planning.

#### Multiple Platforms

Both studies that explored the use of multiple platforms reported on acceptability. Koorts et al [51] reported that 83% of adolescents perceived the intervention content as easy to understand; 93% found the app easy to use; 51% perceived the text messages to be useful; 48% liked the weekly challenges; and 38% liked Facebook videos. In the study by Foster et al [30], participants generally did not like carrying 2 phones and would have preferred to receive the messages on their personal phone, with compensation for extra data and messaging. Despite carrying an extra phone, 71% of participants reported that they found the reminders for appointments and for prenatal vitamins helpful. There were 2 negative reports (out of 14 study participants) that the app became an annoyance over time.

#### Summary of Acceptability

The proportion of study participants who considered digital media useful or helpful ranged from 51% to 95% (mean 69%, 8 studies), easy (to use or to understand the content) ranged from 70% to 95% (mean 82%, 5 studies), and enjoyable ranged from 38% to 100% (mean 78%, 3 studies). The proportion of participants who would recommend the text messages or app to a friend ranged from 82% to 100% (mean 91%, 3 studies). The app and text features that the study participants liked included convenience, short length of the messages, usefulness of the information received, the capacity for interactive dialogue, the ability to create content, and reminders. Negative feedback (which was minimal) included technical issues, lack of interest in the content, not remembering to use the app, not noticing alerts, frequent reminder prompts, and the inconvenience of carrying 2 phones.

### Health Behavior Outcomes

Meta-analysis regarding health behavior outcomes was not completed because of the high degree of heterogeneity among the studies. Of the 18 studies that measured the impact on one or more health behaviors, 15 (83%) studies achieved at least 1 of their health behavior objectives, and 6 (33%) achieved all of their objectives. However, owing to the high prevalence of pilot studies, pretest-posttest design study designs, and relatively small sample sizes (median 77.5), the strength of evidence related to health behavior change was generally low and the risk of bias was moderate to high for the studies in this review (Multimedia Appendices 4 and 5). Additional factors leading to high risk of bias included a lack of valid and reliable data collection tools and high attrition rates. The strength of evidence was, on average, insufficient in the social media studies, low in the texting studies, and low to moderate in the mobile app studies and the studies exploring multiple platforms.

## Discussion

### Principal Findings

This study presents a review of digital communication campaigns used by community-serving agencies to promote health behavior change among vulnerable families. Furthermore, the study reviews their purpose, platform, and key methodologies and systematically describes the engagement with and acceptability of the respective campaigns among this population. Most of the community-serving organizations were WIC agencies, followed by health departments and K-12 schools. The platforms of interest in these studies included SMS text messaging, mobile apps, and social media; and most campaigns promoted healthy eating and PA, breastfeeding, and healthy pregnancies. The results of this review suggest that there is significantly more research focusing on the use of SMS text messaging to promote healthy behaviors in this population than the other 2 platforms and is consistent with prior studies [23]. This could be explained by the relative time and cost efficiency of implementing a texting campaign, the widespread use of texting (compared with the use of social media and mobile apps), and the alignment of texting with existing practices at community-serving agencies (ie, communicating by phone with clients).

The findings of this review related to engagement with and acceptability of these platforms are important considerations for community-serving agencies exploring the use of digital media campaigns to improve their clients’ health. All 3 platforms (9 texting studies, 5 app studies, and 2 social media studies) were highly acceptable, including when combined, with no notable differences in acceptability between the platforms. Study participants appreciated the convenience, short length of the messages (for texting and app studies), usefulness of the information received, and reminders (in texting studies). In addition, the capacity for interactive dialogue when available was particularly appreciated, as was the ability to create content.

Compared with acceptability, the engagement results were less consistent. Engagement was moderate with texting (7/23, 30% studies) and mobile app campaigns (6/23, 26% studies) and low with social media campaigns (3/23, 13% studies). In social media studies, engagement was higher at the beginning of the study period but tapered off over time. Campaigns with tailored messages and opportunities for interaction between study participants and providers tended to elicit greater engagement. This finding is supported by prior research that demonstrates that highly tailored interactive messages (eg, two-way texting and providing opportunities for client feedback) tend to be more successful [13,52,53]. In this review, 9% (2/23) of the studies explored the use of two-way texting for peer support, specifically support from breastfeeding peer counselors [42,43]. Similarly, Dion [29] described the benefits of peer support on a Facebook page used for home visiting services for new mothers. These studies provide further evidence for the high acceptability and appreciation for digital support from peers, which has been demonstrated by Fortuna et al [54] in a 2020 review of digital peer support provided by mental health professionals. Fortuna et al noted that digital support enables asynchronous communication and can facilitate wider reach for peer health professionals.

There was limited evidence to suggest that these digital media campaigns were effective in improving all targeted health behaviors; however, most interventions were successful or partially successful in improving some targeted health behaviors. The strength of evidence was generally low because of the high number of pilot studies, pretest-posttest study designs, and relatively small sample sizes in the study sample. The evidence base could be strengthened by a greater number of randomized controlled trials, as well as studies with larger sample sizes, such as prospective cohort studies. Triantafyllidis et al [55] similarly called for more high-quality studies of mobile health interventions. Considering the COVID-19 pandemic and increased use of digital communication across public health programs, high-quality studies of these platforms are urgently needed.

A key consideration for community organizations implementing digital health campaigns is the need to protect the data security of clients. Web-based consumer data are generally not well protected [7]. The use of mobile apps, self-tracking devices, and social media sites can render people vulnerable to web-based predators. This consideration was not adequately addressed in the studies in this review. Community organizations should take measures to protect their clients’ data privacy, and more research is needed to identify best practices for safeguarding the personal data of health care and social service agency clients.

Another important consideration is the disparities that exist in access to and use of digital communication technology and varying levels of digital health literacy among low-income groups, immigrants to the United States, and people with lower educational attainment (known as the “digital divide”) [17,56-58]. The studies in this sample generally avoided issues related to access by excluding participants without a mobile phone or, in the case of 6 studies, providing a mobile phone with a cellular or data plan to study participants. This could jeopardize the generalizability of the findings related to engagement and acceptability because the sample of this review is likely more digitally connected than the broader population of pregnant women, parents, caregivers, and adolescents.

With regard to digital health literacy, research suggests that one way to help ensure that digital communication campaigns are accessible to people with lower levels of digital health literacy is to actively involve members of the target population in the design of messages and other forms of content [4]. In this review, 9 studies described using participatory designs, including pilot testing, interviews, focus groups, surveys, usability testing, and convening diverse advisory boards. These designs are promising and should be integrated into a greater number of digital health communication studies.

### Limitations

This review has several additional limitations. First, the body of studies was heterogeneous with regard to the study design, study objectives, sample size, and measures of health outcomes. Furthermore, there were no clear definitions of platform engagement in the included studies and very little consistency in how engagement was measured and reported, making it difficult to compare studies even within 1 digital platform. Although the authors were able to summarize the state of the literature, this heterogeneity prevented meta-analysis and made it difficult to generalize the findings. Second, the lack of standardized keywords for the third concept in the literature search may have led to some studies that matched the criteria being excluded inadvertently. Terms such as “mHealth,” “text message interventions,” “social media–based interventions,” “digital technology,” “digital health,” and “digital health and technology intervention” were not included in the search. Future reviews of this subject matter should consider a broader set of search terms. Third, although the authors originally intended to include studies from a gray literature database, the study that met the inclusion criteria was ultimately considered anomalous from the peer-reviewed studies and excluded from the sample. The exclusion of gray literature may have precluded the authors from exploring certain relevant themes, as many of the studies that are conducted by or involve community organizations are less formal and are not reported in peer-reviewed journals.

### Conclusions

This study was the first systematic review of the use of different digital media platforms by community-serving agencies. Digital media platforms are an important component of the health promotion activities of community-serving agencies in high-income English-speaking countries. Although the effectiveness of these strategies in improving health behaviors was inconclusive, low-income pregnant women, parents and caregivers of young children, and adolescents demonstrated moderate levels of engagement with and high levels of acceptability of these strategies.

Higher-quality studies with larger sample sizes are needed to strengthen the evidence base for the use of these platforms to improve health behaviors. Likewise, more research is needed to consistently measure and report participant engagement within and across platforms to discern which platforms and strategies are best suited for specific populations and purposes. This information could be helpful for community-serving agencies in their decision-making regarding which platforms and strategies would best suit their clients’ needs. Finally, future studies should consider strategies for protecting clients’ personal data, and more studies should involve members of the target population in the design of messages and content.

### Additional Information

This review was not registered, and a review protocol was not prepared.

## Appendix

Multimedia Appendix 1 PRISMA (Preferred Reporting Items for Systematic Reviews and Meta-Analyses) 2020 item checklist.

Multimedia Appendix 2 Purpose of digital campaigns.

Multimedia Appendix 3 Study results.

Multimedia Appendix 4 Risk of bias of included studies.

Multimedia Appendix 5 Strength of evidence assessments of included studies.

## Abbreviations

FV: fruit and vegetable
PA: physical activity
PRISMA: Preferred Reporting Items for Systematic Reviews and Meta-Analyses
RAW-PA: Raising Awareness of Physical Activity
WIC: Special Supplemental Nutrition Program for Women, Infants, and Children
