# Supplementary material for: Engagement With and Acceptability of Digital Media Platforms for Use in Improving Health Behaviors Among Vulnerable Families: Systematic Review
Source: J Med Internet Res. 2023 Feb 3;25:e40934. doi: 10.2196/40934 (PMC9938444; doi:10.2196/40934)
Supplement: Multimedia Appendix 2 [file jmir_v25i1e40934_app2.docx]

**Multimedia Appendix 2.** Purpose of digital campaigns

|  | One-way texting | | | | | Two-way texting | | | | | | | Mobile app | | | | | | Social media | | | Multiple | |
| --- | --- | --- | --- | --- | --- | --- | --- | --- | --- | --- | --- | --- | --- | --- | --- | --- | --- | --- | --- | --- | --- | --- | --- |
| Study reference | Evans et al, 2012 | Gazmararian et al, 2014 | Holmes et al, 2020 | Power et al, 2018 | Tagai et al, 2020 | Banna et al, 2017 | Palacios et al, 2018 | Griffin et al, 2018 | Griffin et al, 2020 | Harari et al, 2017 | Martinez-Brockman et al, 2017 | Song et al, 2013 | Clarke et al, 2018 | Gilmore et al, 2017 | Hull et al, 2017 | Nollen et al, 2014 | Reyes et al, 2018 | Zhang et al, 2020 | Allen et al, 2020 | Dion, 2015 | Zhang et al, 2021 | Koorts et al, 2020 | Foster et al, 2015 |
| General health messages |  |  | ✓ | ✓ |  | ✓ | ✓ | ✓ | ✓ | ✓ |  | ✓ | ✓ |  | ✓ | ✓ | ✓ | ✓ | ✓ | ✓ | ✓ | ✓ |  |
| Individually tailored messages | ✓ | ✓ |  |  | ✓ |  |  |  |  |  | ✓ |  |  | ✓ |  |  |  |  |  |  |  |  | ✓ |
| Reminders |  |  |  |  |  |  |  | ✓ | ✓ |  |  |  |  |  | ✓ |  | ✓ |  |  |  |  |  | ✓ |
| Support |  |  |  |  | ✓ |  |  | ✓ | ✓ | ✓ | ✓ | ✓ |  |  |  |  | ✓ |  |  | ✓ |  |  | ✓ |
| Behavior monitoring |  |  |  |  |  |  |  | ✓ | ✓ |  |  |  |  | ✓ |  | ✓ |  |  |  |  |  | ✓ |  |
