# Supplementary material for: Engagement With and Acceptability of Digital Media Platforms for Use in Improving Health Behaviors Among Vulnerable Families: Systematic Review
Source: J Med Internet Res. 2023 Feb 3;25:e40934. doi: 10.2196/40934 (PMC9938444; doi:10.2196/40934)
Supplement: Multimedia Appendix 3 [file jmir_v25i1e40934_app3.docx]

**Multimedia Appendix 3.** Study results

| Digital media platform | Study reference | Results: Engagement with and/or Acceptability of Digital Campaign | Results: Health Behavior Change |
| --- | --- | --- | --- |
| Texting (one-way) | Evans et al, 2012 | Engagement/acceptability not measured | 5% (non-significant) increase in going online to search for prenatal care information. Reported consumption of alcoholic beverages after finding out about their pregnancy decreased from 3.5% at baseline to 1.1% at follow-up. Consumption of >/= 3 servings of fruit/day increased by 3%. Having smoked in last 30 days decreased from 5.8% to 1.2%. Significant increase in agreement with statement “I am prepared to be a new mother”. |
| Texting (one-way) | Gazmararian et al, 2014 | 51% attempted self-enrollment; 69% who attempted successfully enrolled; 92% regularly read all messages; 88% planned to continue enrollment. Few participants (1%) expressed concerns with enrollment; 95% reported it was easy. | Not reported |
| Texting (one-way) | Holmes et al, 2020 | Engagement/acceptability not measured | Mean gestational weight gain was similar in the control group (14.1 ± 11.4 kg) and the intervention group (15.5 ± 11.6 kg). |
| Texting (one-way) | Power et al, 2018 | Parents liked intervention; 79% thought Txt4HappyKids was very credible; 71% found text messages very useful; 67% reported wanting to receive more texts about consuming FV^a^; 82% would recommend the program to a friend; 76% felt they received right number of messages; no one reported receiving too many messages. | 92% of completers reported that since participating in Txt4HappyKids, they served their child more FV. 83% and 78% of completers agreed or strongly agreed, respectively, that more FV were available in their home since participating. |
| Texting (one-way) | Tagai et al, 2020 | Participants expressed high satisfaction and understandability and did not report that program was bothersome. 2/43 participants said program was not helpful, wishing the “messages were more detailed” and “more in-person support would be more beneficial.” | Both relapsers and non-relapsers saw a decrease in distressing events over time, and non-relapsers had an increase in pros vs. cons to quitting. Neither group had any significant changes in knowledge or ways of quitting. |
| Texting (two-way) | Banna et al, 2017 | 70% of participants said SMS was sent at acceptable times, useful and not irritating, and easy to understand. Participants enjoyed the experience of participating. Participants most liked convenience followed by short length of messages and usefulness of information. Most participants reported not having any problems receiving or responding to messages. Response to SMS was greater on average for first 4 questions (52-55%) than for last 3 (32-34%). | See Palacios et al, 2018 |
| Texting (two-way) | Palacios et al, 2018 | See Banna et al, 2017 | Feeding practices did not differ between groups at end of study. However, there was a trend (close to significant, *P* value 0.07) for a greater proportion of caregivers in the intervention group stopping the feeding when infants showed signs of being full (44.1%) compared to control (39.5%). No significant group differences were found in weight status at the end of the trial and with weight changes from visit 1 to visit 2. |
| Texting (two-way) | Griffin et al, 2018 | 58% said intervention was helpful, and most said number of texts was about right. Participants found texts informative and motivating. | Significant reduction in mean body weight and mean BMI^b^ in those who completed both assessments. Participants lost an average of 4.7 lbs. and reduced BMI by 0.8 kg/m.2. Significant improvement in goal setting, physical activity behaviors, sedentary behaviors, and environmental factors. |
| Texting (two-way) | Griffin et al, 2020 | 56 participants completed program. 39 had text responses for weekly step counts at pre- and postintervention. Over 12 weeks, participants received a total of 4,704 text response prompts for daily step counts and a total of 672 text response prompts for body weight. At postintervention, 3,150 participant step count text responses and 482 body weight text responses were received. Average of 8.61 ±3.4 body weight responses per participant were received (mode = 9). | Step counts increased significantly (mean increase 1689 ± 689). Significant increase in participants who knew how to set goals to increase PA, planned to engage in vigorous-intensity exercise, planned to engage in moderate-intensity exercise, exercised 30 minutes on previous day, and knew number of steps walked on previous day. Body weight decreased significantly (mean decrease 3.99 ± 12.7 lbs.). |
| Texting (two-way) | Harari et al, 2017 | 83% of participants reported they “always” or “very often” read LATCH^c^ texts prenatally; 80% always or very often read texts post‐partum; 90% texted an original comment or question (other than “ok” or “thanks”); 77% clicked on at least one web link; 91% reported they would recommend the texting program to a friend. | Exclusive breastfeeding prevalence at 2 weeks post‐partum in intervention group was 50% vs. 31.8% in control arm (not significant). Contact between mothers and peer counselors within 48 hours of delivery was significantly higher in intervention group: 86.6% vs. 27.3% control. Mothers in intervention group reported meeting their breastfeeding goals more frequently than mothers in the control arm, 96.1% vs. 73.6% (close to significance, *P* value 0.06). |
| Texting (two-way) | Martinez-Brockman et al, 2017 | Participants responded to an average of 27% of text messages across prenatal and postpartum periods. Participants clicked on an average of 22% of possible videos, weblinks, and photos. | Correlations were weak or moderate, with a few exceptions. Action self-efficacy and intentions were highly correlated, as were maintenance and recovery self-efficacy. Action self-efficacy significantly predicted intention to exclusively breastfeed. Planning, maintenance self-efficacy, and recovery self-efficacy each significantly predicted exclusive breastfeeding behavior. |
| Texting (two-way) | Song et al, 2013 | 291 participant questions received. 80% reported it was easy to text questions; 65% reported information was easy to understand; 65% reported texting system made finding information quick and easy. Participants liked the fact that system allowed for interactive dialogue. | 78.9% of participants agreed or strongly agreed that they were better prepared for labor and delivery and knew how to have a healthy baby. 60% agreed or strongly agreed that using program helped them think of more questions to discuss with health care providers and feel more prepared to see health care provider. 88.9% agreed or strongly agreed that they searched for pregnancy related information and/or paid more attention to it. Use of program significantly reduced depression and perceived stress and improved mental well-being. |
| Mobile apps | Clarke et al, 2018 | Most cooks created 5-10 Veggie-Books and 1-4 SecretBooks. Most children created 0-5 VeggieBooks and 0-1 SecretBooks. When planning meals or using VeggieBooks in their kitchens, experimental participants almost evenly split in their preference for print or phone screen content. | Average number of unique preparations with target vegetables higher for experimental (median of means 4.17 vs. 3.03). All 6 control sites declined in using 24 vegetables, whereas 6/9 experimental sites increased average vegetable usage. Among families with app, 29% of staff conversations contained mentions their families were eating and/or enjoying more vegetables vs. only 1% of control conversations. |
| Mobile app | Gilmore et al, 2017 | Adherence involved number of days participants weighed themselves and number of days step counts were recorded using app. Low adherence was defined as 1-3 days/week of engagement, medium adherence was 3-5 days/week, and high adherence was 5 or more days/week. 37% were considered low adherence, 37% were medium, and 26% were high. | No significance in weight change, blood pressure, or waist/hip ratio between intervention and control groups. High adherence group had significant reduction in body weight and percent body fat. |
| Mobile app | Hull et al, 2017 | Participants used app on average once/week for approximately 4.5 minutes/session. 9/10 mothers reported using at least 1 of the 3 app components. Qualitative feedback from non-users pointed to several barriers including technical difficulties using app, lack of interest in content, not remembering to use it, and not noticing alerts. | Participants on average agreed that there were benefits of using the app. |
| Mobile app | Nollen et al, 2014 | Girls used program on 63% of days, responded to 42% of prompts, and earned an average of 23.9 songs. Favorite parts of the program were obtaining songs (68.2%) and setting goals (36.4%). Least favorite part was the reminder prompts (31.8%). | Significant association between intervention and SSB^d^ intake. Girls responding to more prompts showed greater reductions in SSB compared with those responding to fewer prompts (mean difference -0.31 daily servings). No significant associations between intervention and FV intake, screen time-related behaviors, or BMI. |
| Mobile app | Reyes et al, 2018 | All 14 participants stated they would recommend app to their friends and families and thought app would be good for women of all ages and women like themselves. All 14 thought app was easy to use, liked using app, and had no privacy concerns or technical difficulties. 9/14 participants thought app was “simple” or “self-explanatory.” 5/9 reached at follow-up used app. | Not reported |
| Mobile app | Zhang et al, 2020 | 72.3% of households used app at least once during study period. App users actively used app in 7.9 benefit cycles, equivalent to 85.8% of all benefit cycles in study period. App users activated the app once a week. | App users had a higher WIC^e^ redemption rate than non-users (significant in almost all food categories). Having 1 additional active app cycle was associated with 0.4 to 2% increase in redemption rates; increasing active cycle rates by 10% was associated with 0.9 to 4.7% increase. Number of days of app usage was significantly positively associated with redemption rates. |
| Social media | Allen et al, 2020 | 36% checked Twitter account daily; 45% checked Twitter account every few weeks or less. 6 participants liked tweets and 4 retweeted. 2/35 women (5.7%) ‘blocked’ the tweets. 71% agreed with statement “Twitter messages are a good way to educate women about the HPV^f^ vaccine.” | There was no statistically significant change in HPV knowledge, vaccination decision self-efficacy, or intent to be vaccinated in next 6-12 months. Participants perceived high benefits to HPV vaccination in terms of vaccine efficacy (mean of 9 on scale of 0–12) and vaccine safety (mean of 6 on a scale of 0–9); moderate barriers to HPV vaccine uptake (mean of 4 on scale of 1–8). |
| Social media | Dion, 2015 | Use of Facebook was appreciated by clients and improved engagement with service. 61% of mothers who had a baby in past year joined Facebook page. 23 clients responded with "likes" and several provided positive comments when asked how satisfied they were with page. | Not reported |
| Social media | Zhang et al, 2021 | Recipes were most-liked content on Facebook page among new followers, followed by tips on feeding children and tips on food planning. | No statistically significant difference between self-reported healthy eating outcomes except feeding healthy meals on a budget (p = 0.02). 33% rated page as impacting them “a great deal,” and 25% rated the impact as “somewhat.” |
| Multiple: Mobile app, social media | Koorts et al, 2020 | 83.3% of adolescents perceived intervention content as easy to understand, 93.3% found app easy to use, 50.8% perceived text messages to be useful, 47.5% liked weekly challenges, and 38.3% liked Facebook videos. 85% of adolescents registered for Facebook group. Engagement in Facebook group declined over time (only 18.6% self-reported using app daily post-intervention). Teachers perceived app as highly acceptable at school level. | 70.8% of adolescents perceived that app increased their PA^g^ motivation, and 78.2% perceived that it increased their PA awareness. 34.5% of adolescents perceived that program challenges motivated them to be more active, 61.7% perceived that program encouraged them to increase activity on their own, 40.3% perceived that program encouraged them to increase activity with family, and 54.2% perceived that program encouraged them to increase activity with friends. |
| Multiple: Mobile app, texting (two-way) | Foster et al, 2015 | Participants did not like carrying 2 phones and preferred to receive messages on personal phone, with compensation for extra data and messaging. 10/14 reported reminders for appointments and prenatal vitamins helpful. 2 negative reports that app became an annoyance over time. Average response rates to texts ranged from 0 to 100% depending on message and phase and trended down over time. | Not reported |

^a^FV: fruits and vegetables

^b^BMI: body mass index

^c^LATCH: Lactation Advice thru Texting Can Help

^d^SSB: Sugar-sweetened beverage

^e^WIC: Special Supplemental Nutrition Program for Women, Infants, and Children

^f^HPV: Human Papilloma Virus

^g^PA: physical activity.
