# Supplementary material for: Engagement With and Acceptability of Digital Media Platforms for Use in Improving Health Behaviors Among Vulnerable Families: Systematic Review
Source: J Med Internet Res. 2023 Feb 3;25:e40934. doi: 10.2196/40934 (PMC9938444; doi:10.2196/40934)
Supplement: Multimedia Appendix 4 [file jmir_v25i1e40934_app4.docx]

**Multimedia Appendix 4.** Risk of bias assessments of included studies

| Digital platform | Study reference | Selection bias | Study design | Confounders | Data collection methods | Withdrawals and drop-outs | Overall risk of bias (ROB) (Strong: Low ROB) |
| --- | --- | --- | --- | --- | --- | --- | --- |
| Texting | Banna et al, 2017 | Moderate | Strong | Weak | Strong | Strong | Moderate |
|  | Evans et al, 2012 | Strong | Strong | Strong | Strong | Moderate | Strong |
|  | Gazmararian et al, 2014 | Moderate | Moderate | Strong | Moderate | Weak | Moderate |
|  | Griffin et al, 2020 | Weak | Weak | Strong | Moderate | Weak | Weak |
|  | Griffin et al, 2018 | Weak | Weak | Strong | Strong | Weak | Weak |
|  | Harari et al, 2017 | Moderate | Strong | Strong | Weak | Strong | Moderate |
|  | Holmes et al, 2020 | Moderate | Strong | Strong | Moderate | Strong | Strong |
|  | Martinez-Brockman et al, 2017 | Strong | Moderate | Weak | Strong | Moderate | Moderate |
|  | Palacios et al, 2018 | Strong | Strong | Strong | Moderate | Strong | Strong |
|  | Power et al, 2018 | Weak | Weak | Strong | Strong | Moderate | Weak |
|  | Song et al, 2013 | Weak | Weak | Strong | Weak | Strong | Weak |
|  | Tagai et al, 2020 | Moderate | Weak | Strong | Moderate | Weak | Weak |
| Apps | Clarke et al, 2018 | Strong | Strong | Strong | Strong | Weak | Moderate |
|  | Gilmore et al, 2017 | Strong | Moderate | Strong | Moderate | Strong | Strong |
|  | Hull et al, 2017 | Moderate | Weak | Strong | Weak | Moderate | Weak |
|  | Nollen et al, 2014 | Moderate | Moderate | Strong | Moderate | Moderate | Strong |
|  | Reyes et al, 2018 | Moderate | Weak | Strong | Moderate | Moderate | Moderate |
|  | Zhang et al, 2020 | Strong | Weak | Strong | Moderate | Moderate | Moderate |
| Social media | Allen et al, 2020 | Moderate | Weak | Moderate | Strong | Strong | Moderate |
|  | Dion, 2015 | Moderate | Weak | Moderate | Weak | Moderate | Weak |
|  | Zhang et al, 2021 | Moderate | Weak | Strong | Moderate | Weak | Weak |
| Multiple | Foster et al, 2015 | Moderate | Weak | Strong | Weak | Strong | Weak |
|  | Koorts et al, 2020 | Strong | Moderate | Strong | Weak | Strong | Moderate |
