# Supplementary material for: Engagement With and Acceptability of Digital Media Platforms for Use in Improving Health Behaviors Among Vulnerable Families: Systematic Review
Source: J Med Internet Res. 2023 Feb 3;25:e40934. doi: 10.2196/40934 (PMC9938444; doi:10.2196/40934)
Supplement: Multimedia Appendix 5 [file jmir_v25i1e40934_app5.docx]

**Multimedia Appendix 5.** Strength of evidence assessments of included studies

| Digital platform | Study reference | Risk of bias | Directness | Consistency | Precision | Reporting bias | Overall strength of evidence |
| --- | --- | --- | --- | --- | --- | --- | --- |
| Texting | Banna et al, 2017 | Moderate | Direct | Inconsistent | Imprecise | Undetected | Low |
|  | Evans et al, 2012 | Moderate |  |  |  |  |  |
|  | Gazmararian et al, 2014 | Moderate |  |  |  |  |  |
|  | Griffin et al, 2020 | High |  |  |  |  |  |
|  | Griffin et al, 2018 | High |  |  |  |  |  |
|  | Harari et al, 2017 | Moderate |  |  |  |  |  |
|  | Holmes et al, 2020 | Moderate |  |  |  |  |  |
|  | Martinez-Brockman et al, 2017 | Moderate |  |  |  |  |  |
|  | Palacios et al, 2018 | Low |  |  |  |  |  |
|  | Power et al, 2018 | High |  |  |  |  |  |
|  | Song et al, 2013 | High |  |  |  |  |  |
|  | Tagai et al, 2020 | High |  |  |  |  |  |
| Apps | Clarke et al, 2018 | Moderate | Direct | Inconsistent | Imprecise | Undetected | Low-moderate |
|  | Gilmore et al, 2017 | Low |  |  |  |  |  |
|  | Hull et al, 2017 | High |  |  |  |  |  |
|  | Nollen et al, 2014 | Moderate |  |  |  |  |  |
|  | Reyes et al, 2018 | Moderate |  |  |  |  |  |
|  | Zhang et al, 2020 | Moderate |  |  |  |  |  |
| Social media | Allen et al, 2020 | Moderate | Indirect | Inconsistent | Imprecise | Undetected | Insufficient |
|  | Dion, 2015 | High |  |  |  |  |  |
|  | Zhang et al, 2021 | High |  |  |  |  |  |
| Multiple | Foster et al, 2015 | High | Direct | Inconsistent | Imprecise | Undetected | Low-moderate |
|  | Koorts et al, 2020 | Moderate |  |  |  |  |  |
